# Neuroprotective Effects of Marine Algae

**DOI:** 10.3390/md9050803

**Published:** 2011-05-10

**Authors:** Ratih Pangestuti, Se-Kwon Kim

**Affiliations:** 1 Marine Biochemistry Laboratory, Department of Chemistry, Pukyong National University, Busan 608–737, Korea; E-Mail: ratihpangestuti@pknu.ac.kr; 2 Marine Bioprocess Research Center, Pukyong National University, Busan 608–737, Korea

**Keywords:** marine algae, neuroprotective, neuroprotection

## Abstract

The marine environment is known as a rich source of chemical structures with numerous beneficial health effects. Among marine organisms, marine algae have been identified as an under-exploited plant resource, although they have long been recognized as valuable sources of structurally diverse bioactive compounds. Presently, several lines of studies have provided insight into biological activities and neuroprotective effects of marine algae including antioxidant, anti-neuroinflammatory, cholinesterase inhibitory activity and the inhibition of neuronal death. Hence, marine algae have great potential to be used for neuroprotection as part of pharmaceuticals, nutraceuticals and functional foods. This contribution presents an overview of marine algal neuroprotective effects and their potential application in neuroprotection.

## 1. Introduction

Ninety percent of the world’s living biomass is found in the oceans with marine species comprising approximately half of the total global biodiversity [1,2]. This wide diversity of organisms is being recognized as a reservoir of potent molecules which are elicited by marine organisms to help them survive in the hostile environment [2,3]. Among marine organisms, marine algae have been identified as an under-exploited plant resources [4,5]. The term marine algae, as used herein, generally refer to marine macroalgae or sometimes referred to as seaweeds.

Marine algae can be classified into three classes based on their pigmentation, namely brown, red, and green algae, which are referred to as Phaeophyceae, Rhodophyceae, and Chlorophyceae, respectively [6]. Since the 1940s, production of algal polysaccharides has attained commercial significance through their application as thickening and gelling agents for various industrial applications [7]. Moreover, marine algae are recognized as rich sources of structurally diverse biologically active compounds with great pharmaceutical and biomedical potential. Researchers have revealed that marine algal originated compounds exhibit various biological activities such as anticoagulant [8,9], anti-viral [10,11], antioxidant [12–14], anti-allergic [15], anti-cancer [16], anti-inflammatory [17], anti-obesity [18–20], *etc*. Furthermore, several scientific studies have provided insight into neuroprotective properties of marine algae. Many species of marine algae have long been used in food diets as well as traditional remedies in Eastern countries and more recently in Europe and America. Hence, marine algae have great potential to be used in neuroprotection [21].

In recent years, biological activities, nutritional value, and potential health benefits of marine algae have been intensively investigated and reviewed. This review, however, focuses specifically on the neuroprotective effects of marine algae and emphasizes their potential application as future pharmaceutical candidates to prevent neurodegenerative diseases.

## 2. Bioactivities and Neuroprotective Effects of Marine Algae

### 2.1. Antioxidant

Oxidative stress is the result of an imbalance between pro-oxidant and antioxidant homeostasis that leads to the generation of toxic reactive oxygen species (ROS) [22]. Compared to other parts of our body, the central nervous system (CNS) is more sensitive to oxidative stress due to its high oxygen consumption and lipid content. Increased oxidative stress in the CNS will further lead to lipid peroxidation, DNA and protein damage [23]. Oxidative stress in the CNS has been demonstrated to involve excitotoxicity and apoptosis, the two main causes of neuronal death. Furthermore, oxidative stress has also been implicated the progression of Alzheimer’s disease (AD), Parkinson’s disease (PD), multiple sclerosis (MS) and other neurodegenerative diseases [24,25]. Antioxidants may have a positive effect in the CNS and seem to be a promising approach of neuroprotection therapy, as they can protect the CNS against free radical mediated oxidative damage [26]. However, our endogenous antioxidant defenses are not always completely effective and exposure to damaging environmental factors is increasing, therefore it seems reasonable to propose that exogenous antioxidants could be effective in diminishing the cumulative effects of oxidative damage. Presently, antioxidants constitute a major component of clinical and experimental drugs that are currently considered for prevention of neurodegenerative diseases and therapy [27].

Antioxidant activities of marine algae have been determined by various methods such as 1,1-diphenyl-2-picryl hydrazyl (DPPH) radical scavenging, 2,2′-azinobis-3-ethylbenzo thizoline-6-sulphonate (ABTS) radical scavenging, singlet oxygen quenching activity, lipid peroxide inhibition, superoxide and hydroxyl radical scavenging assays. Lim *et al.* demonstrated that *Neorhodomela aculeate*, which is also known as *Rhodomela confervoides*, was able to scavenge DPPH with an IC_50_ = 90 μg/mL and at a concentration of 20 μg/mL completely suppressed H_2_O_2_ induced lipid peroxidation in rat brain homogenate [28]. Furthermore, Fallarero *et al.* showed that *Halimeda incrassata* and *Bryothamniom triquetrum* are potent ROS scavengers in mouse hypothalamic (GT1–7) cells [29]. Novoa *et al.* reported that the antioxidant and ROS scavenging activity of *B. triquetrum* are related to their high phenolic contents [30]. Dieckol, a phenolic compound isolated from brown algae has been shown to scavenge ROS production in murine microglia (BV2) cells [31]. Wijesekara and Kim reported that most phenolic compounds which were purified from marine algae are responsible for marine algal antioxidant activities and protective effects against oxidative stress induced cell damage [32]. Phenolic compounds act as free radical scavengers, reducing agents and metal chelators, and thus effectively inhibit lipid oxidation. In addition, Yan *et al.* demonstrated that carotenoids have a strong radical scavenging activity and are found as a major antioxidant in marine algae [33,34]. Young and Lowe indicated that structure, physical form, location or site of action, potential interaction with another antioxidant, concentration and partial pressure to oxygen may affect the antioxidant activities of carotenoids in biological systems [35]. Fucoxanthin obtained from *Padina tetrastromatic* has shown higher potential to be used as an antioxidant than β-carotene in modulating antioxidant enzyme in plasma and liver of retinol deficient rats [36,37]. However, the exact mechanisms of action how fucoxanthin exerts antioxidative effect in rat induced by retinol deficiency are not yet completely understood. Moreover, the cytoprotective effect of fucoxanthin against ROS formation induced by H_2_O_2_ in monkey kidney fibroblast (Vero) cells has been observed [38]. Two hydroxyl groups present in the ring structure of fucoxanthin may correlate to the inhibition of ROS formation. Indeed, it has been reported that the number of hydroxyl groups on the ring structure is correlated with the effects of ROS suppression. Moreover, it has also been shown that some marine algal sulfated polysaccharides (SPs) can be used as potent antioxidants [39,40]. Antioxidant activity of marine algal SPs depends on their structural features such as degree of sulfating, molecular weight, type of the major sugar and glycosidic branching [41,42]. However, bioactivities of marine algal carotenoids and SPs against oxidative stress in the CNS have not been demonstrated yet.

Based on those findings, it can be suggested that among various organisms in the marine environment, marine algae prove to be one of the useful candidates that can protect the CNS against oxidative degradation. Hence, developing novel molecules derived from marine algae which promote antioxidant activity in the CNS may lead to the development of effective neuroprotective agents. Furthermore, it is also important to determine whether antioxidants derived from marine algae can be used as prophylactic neuroprotective agents in order to slow down the progression of neurodegenerative diseases in populations that are at high risk, such as the elderly. Additionally, antioxidant activities of marine algal carotenoids, SPs and other bioactive compounds in the CNS warrant further investigations.

### 2.2. Anti-Neuroinflammation

Inflammation has been found to be the pathophysiological mechanism underlying many chronic diseases such as cardiovascular disease, diabetes, certain cancers, arthritis, and neurodegenerative diseases [43]. Recent studies demonstrated that resulting production of inflammatory responses and neurotoxic factors in the CNS is sufficient to induce neurodegeneration in a rat model [44]. Several cell types have been demonstrated as contributors in inflammation-mediated neurodegeneration, yet microglia are implicated as critical components of the immunological insult to neurons [45]. Microglia are the immune cells in the CNS, they enters the system from the blood circulation early in an organism’s development and serve a role of immune surveillance [43]. Ramified or resting microglia constitute 5–20% of glial populations in the CNS [46]. Recent study demonstrated that activation of microglia and the resulting production of pro-inflammatory and neurotoxic factors are sufficient to induce neurodegeneration in a rat model. Furthermore, activation of microglia and excessive amounts of pro-inflammatory mediators release by microglia have been observed during the pathogenesis of PD, AD, MS, AIDS dementia complex, as well as post neuronal death in cerebral stroke and traumatic brain injury [44,47]. Therefore, a mechanism to regulate inflammatory response release by microglia may have important therapeutic potential for the treatment of neurodegenerative diseases.

Numerous studies has documented anti-inflammatory activities of marine algae *in vitro* and *in vivo* [48]. However, scientific analysis of anti-neuroinflammatory activity of marine algae has been poorly carried out and until now only few studies were reported. *Ecklonia cava* (Phaeophyceae; Laminareaceae), also known as “sea trumpet”, has been reported to possess anti-inflammatory activity [49–51]*. E. cava* was able to suppress the levels of pro-inflammatory mediators such as nitric oxide (NO), prostaglandine-E_2_ (PGE_2_) and pro-inflammatory cytokines (tumor necrosis factor-α (TNF-α), interleukin-6 (IL-6) and interleukin-1β (IL-1β)) in lipopolysaccharides (LPS)-stimulated BV2 cells by blocking nuclear factor-κB (NF-κB) and mitogen-activated protein kinases (MAPKs) activation [31,51]. Furthermore, *N. aculeate* decreased NO production and inhibiting inducible NO synthase (iNOS) expression in interferon-gamma (IFN-γ) stimulated BV2 cells [28]. A number of bromophenols have been previously isolated from *N. aculeate* and may be potential anti-neuroinflammatory candidates [52–55]. Another study conducted by Cui *et al.* [56] provide the first evidence that fucoidan isolated from *Laminaria japonica* has a potent inhibitory effect against LPS-induced NO production in BV2 cells. In their study, the average molecular weight of fucoidan was 7000 Dalton, consisting of 48% total sugar (including 28% fucose) and 29% sulfate. Fucoidan at a concentration of 125 μg/mL, significantly inhibited NO production to 75% [56]. NO is a cytotoxic, short lived highly diffusible signaling molecule [57]. A number of studies demonstrated that NO generated by iNOS causes injury and cell death of neuron and oligodendrocytes in the CNS, hence NO is implicated in pathogenesis of various neurodegenerative disease [57,58]. Anti-neuroinflammatory activity of another marine algae species, *Ulva conglobata* has been reported. *U. conglobata* methanolic extracts were able to suppress the expression of pro-inflammatory enzymes, iNOS and cyclooxygenase-2 (COX-2), which accounted for the large production of NO and PGE_2_, respectively [59,60]. Among other mediators released by microglia, NO and PGE_2_ are the main cytotoxic mediators participating in the innate response in the CNS [61,62]. Pro-inflammatory mediators have been found to be elevated in the brain of early AD [63]. For this reasons, agents that inhibit the production of pro-inflammatory mediators have been previously considered as potential candidates for the treatment of neurodegenerative diseases.

Epidemiological studies show that application of non-steroidal anti-inflammatory drugs (NSAIDs) reduces the risk and delays the onset of inflammation in the CNS which further participates in the pathogenesis of some neurodegenerative diseases. NSAIDs mainly act by inhibiting the production of pro-inflammatory mediators. Hence, attenuation of pro-inflammatory mediators in microglia by marine algae demonstrates its potential neuroprotective activity. Furthermore, marine algae as potential anti-neuroinflammatory agents have a great potential application in the pharmaceuticals area as well as the food industry. There are numerous advantages of marine algae use in pharmaceuticals and functional foods, such as relatively low production costs, low cytotoxicity, safety and wide acceptability. However, further studies are needed with clinical trials for marine algal anti-neuroinflammatory activity.

### 2.3. Cholinesterase Inhibitory Activity

Alzheimer’s diseases (AD) is an irreversible, progressive neurodegenerative disease, which resulting in memory loss, behavior disturbances, personality changes and a decline in cognitive abilities [64]. It was stated in the cholinergic hypothesis, that a serious loss of cholinergic function in the CNS contributes significantly to the cognitive symptoms associated with AD [65]. In accordance, neuropathological studies demonstrated that AD was associated with deficiency in the brain neurotransmitter acetylcholine (ACh) [66]. The inhibition of acetylcholinesterase (AChE) enzyme, which catalyzes the breakdown of ACh, may be one of the most realistic approaches to the symptomatic treatment of AD [67]. Presently, a variety of plants has been reported to possess AChE inhibitory activity. *Huperzia serrata*, a Chinese terrestrial herb has been demonstrated to be a potent AChE inhibitor [68]. In addition, Houghton *et al.* reported cholinesterase (ChE) inhibitory activity of *Crinum jagus* and *Crinum glaucum*, two Nigerian *Crinum* species [69]. A number of studies have recently shown AChE inhibitory activity of several marine algae species. A list of marine algae reported to have significant AChE inhibitory activity is presented in Table 1.

Recently, Myung *et al.* reported that dieckol and phlorofucofluoroeckol possess memory enhancing and AChE inhibitory activity [74]. Furthermore, Yoon *et al.* screened ethanolic extracts of 27 Korean marine algae, for inhibitory activity on AChE, and found that extracts from *Ecklonia stolonifera* showed significant inhibitory activity [72]. Two sterols and eight phlorotannins were isolated from *E. stolonifera*. Eckol, dieckol, 2-phloroeckol and 7-phloroeckol demonstrated selective dose dependent inhibitory activities toward AChE; whereas, eckstolonol and phlorofucofuroeckol-A exhibited inhibitory activities toward both AChE and butyrylcholinesterase (BChE). However, phloroglucinol, which is a monomer, and triphlorethol-A, the opened-chain trimer of phloroglucinol, did not inhibit the cholinesterase (ChE) at the concentrations tested. The exact mechanisms underlying this phenomenon have not yet been identified. However, the possible relation between structure of phlorotannins and AChE inhibitory activity has been reported, it is suggested that phlorotannins as polymers of phloroglucinol have appropriately bulky structures, which is then able to mask the ChE and prevents the binding of the substrates. Moreover, as the phloroglucinol monomer and open-chain trimer of phloroglucinol were not able to inhibit the ChE activity, it may suggest that that degree of polymerization and closed-ring structure of phlorotannins play key roles in the inhibitory potential of phlorotannins toward the ChE [72]. In addition, *Hypnea valentiae* and *Ulva reticulate*, two marine algae species from Tamil Nadu, India, also reported to inhibit both AChE and BChE activity [71]. A good balance between AChE and BChE activity has been reported to result in higher efficacy for the treatment of AD [75]. BChE are considered to play a minor role in regulating brain AChE levels. Notably, AChE and BChE mixed inhibition have been found in tacrine and physostigmine, which are licensed drugs used in the treatment of AD.

Taken together, marine red, brown and green algae have potential to be use as functional neuroprotective agents due to their effectiveness in inhibiting ChE activity. Furthermore, some compounds derived from marine algae provided mixed type ChE (AChE and BChE) inhibitory activities, which have been considered to be more effective in the treatment of AD. Some AChE synthetic commercial drugs are known to produce side effects. Hence, researchers have a great interest to study natural herbs that can act as AChE inhibitors. Many kinds of marine algae, consumed for centuries in East Asia countries, are well tolerated and lack harmful side effects. Interestingly, several marine algae species have also been demonstrated as potential AChE inhibitors. Hence, AChE inhibitory activity of marine algae should be screened and further studies with clinical trials are also needed.

### 2.4. Inhibition of Neuronal Death

**Figure 1.**
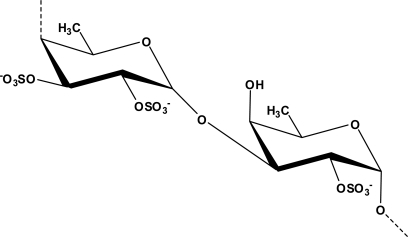
Chemical structure of fucoidan isolated from *Fucus vesiculosus* (Adapted from [39]).

A common pathological hallmark of various neurodegenerative diseases is the loss of particular subsets of neurons [76]. Neurodegeneration of these neural subsets may be a consequence of various forms of neural cell death, including necrosis and apoptosis [77]. A study carried out by Jhamandas *et al.* successfully showed that fucoidan isolated from *Fucus vesiculosus* (Figure 1), was able to protect rat cholinergic neuronal death induced by Aβ_1–42_ [78]. Fucoidan pretreatment blocked the activation of caspase-9 and caspase-3. Caspase-9 and caspase-3 have been suggested to mediate the terminal stages of neuronal apoptosis [79]. Caspase-9 and caspase-3 are two of several central components of the machinery responsible for apoptosis. Therefore, the ability of fucoidan to block the activation of caspase-9 and caspase-3 suggest that inhibition of neuronal death by fucoidan mainly occurs through apoptotic inhibition. In neurodegenerative diseases, apoptosis might be pathogenic, and targeting this process might mitigate neurodegenerative diseases [80]. Furthermore, aqueous extracts of *B. triquetrum* has been demonstrated to protect GT1–7 cells death produced by severe (180 min) chemical hypoxia/aglycemia insult, which further reduced the cytotoxicity and early production of free radicals. The protection exerted by *B. triquetrum* extract seems to be linked to its ability to reduce free-radical generation [81]. The authors suggest that the protective effects of *B. triquetrum* extract are partially related to the presence of ferulic acid [81].

### 2.5. Antineurotoxicity

Neurotoxins are a varied groups of compounds, whose highly specific effects on the nervous system of animals, including humans, is by interfering with nerve impulse transmission [82]. They are able to produce neuronal damage or neurodegeration when administered *in vivo* or *in vitro* [83]. As an example, β-amyloid (Aβ) peptides have been demonstrated to possess neurotoxic effect on neuron and glial cells although the precise mechanisms by which this occurs have yet to be elucidated [84]. Excessive accumulation of Aβ in the brain has been characterized as a major pathological hallmark of AD and recently, fucoidan has been reported to block Aβ neurotoxicity in neuronal cell [78]. Fucoidan treatment abolished the inhibitory effect of Aβ on the phosphorylation of protein kinase C (PKC) which has been demonstrated to stimulate the survival of neurons and prevents Aβ neurotoxicity. PKC causes GSK-3β inactivation and this inactivation in turn leads to the accumulation of cytoplasmic β-catenin and the subsequent translocation of β-catenin to the nucleus, causing TCF/LEF-1-dependent transcriptional activation of growth and differentiation related genes, which is required to stimulate neuronal survival [85]. In addition, Luo *et al.* showed that fucoidan isolated from *L. japonica* was able to protect against 1-methyl-4-phenyl-1,2,3,6-tetrahydropyridine (MPTP)-induced neurotoxicity in animal model of Parkinsonism (C57/BL mice) and dopaminergic (MN9D) cells [86]. The mechanisms of protection provided by fucoidan may partly relate to its antioxidative activity. Furthermore, the results of those studies suggest potential application of fucoidan for PD prevention and or treatment. Moreover, the possible roles of alginates to protects human neuronal (NT2) cells against H_2_O_2_-induced neurotoxicity have previously been demonstrated [87]. *H. incrassata* and *B. triquetrum* at a concentration of 0.2 mg/mL has been shown to protect methyl mercury-induced neurotoxicity in GT1–7 cells [29]. Collectively, marine algae and its bioactive compounds can be used for the development of new generation therapeutic neuroprotective agents against neurotoxins in the CNS.

### 2.6. Other Neuroprotective Activities

Neurite outgrowth is a fundamental neuronal feature and plays an important role in neuronal development during embryogenesis and in the adult brain [88]. *Sargassum macrocarpum* and its two active component, sargaquinoic acid and sargachromechanol, have been shown to promote neurite outgrowth in rat pheochromocytoma (PC12) cells [89–91]. Structure and neurite outgrowth promoting relationship of sargaquinoic acid has been reported by Tsang *et al.* [92]. They reported that quinone is the structural moiety of the sargaquinoic acid molecule which is responsible for the neurite outgrowth-promoting activity. Notably, the hydroxyl group bonded to quinone had a significant effect on neuritogenic activity. In addition, pheophytin a, a chlorophyll-related compound and its analog, vitamin B12 derived from *Sargassum fulvellum* also has potential neurite outgrowth-promoting activity [93,94].

Phlorotannins derived from *Eisenia bicyclis* have been demonstrated to inhibit β-amyloid cleavage enzyme (BACE-1) activity [95]. BACE-1 represents candidate biomarkers of AD, since it initiates the formation of Aβ [96]. When considering that almost all currently available medications for AD are AChE inhibitors, suppression of BACE-1 by phlorotannins will enhance the medications and or therapy for AD patients.

In addition, Lee *et al.* demonstrated that fucoidan treatment resulted in an increase in cell proliferation of human neuroblastoma (SH-SY5Y) cell induced by Aβ [97]. Hence, it may suggest that fucoidan has potential neuroprotective effects.

## 3. Prospects of Marine Algae as Neuroprotective Agents

Neurodegenerative diseases are estimated to surpass cancer as the second most common cause of death among elderly by the 2040s [98,99]. For this reason, a great deal of attention has been expressed by scientists regarding safe and effective neuroprotective agents. Many categories of natural and synthetic neuroprotective agents have been reported. However, synthetic neuroprotective agents are believed to have certain side effects such as dry mouth, tiredness, drowsiness, sleepiness, anxiety or nervousness, difficulty to balance, *etc.* [100]. Hence, nowadays researchers have an interest in studying natural bioactive compounds that can act as neuroprotective agents. Marine algae represent one potential candidate neuroprotective agent. However, development of marine algae as neuroprotective agents still faces several challenges. The rationale for marine algal neuroprotective effects treatment in the CNS is based on established observations and experiments *in vitro* or in animal models only. Up to now, none of the marine algal neuroprotective effects have been examined in human subjects. Therefore, small clinical studies and further large-scale controlled studies are needed. Another important challenge in the development of marine algae as neuroprotective agents is that many drugs failed to provide real neuroprotection in practice. Potential reasons for this failure include inappropriate use of specific neuroprotection/s for a given disease or stage of disease progression or the use of suboptimal doses [101]. Hence, future studies are needed focusing on the synergistic benefits of consuming different marine algae species, recommended doses and timing of intake, and preparation methods for marine algal bioactive compounds in order to maximize the desired protective effect in the prevention of neurodegenerative diseases.

It has been reported that neurodegenerative diseases in East Asian countries were lower than in Europe (*p* < 0.0004) [102,103]. Many studies have indicated potential health benefits of marine algae consumption [7,104]. Thus, lower incidence of neurodegenerative diseases in East Asia may correlate to high fish and marine algae consumption by East Asian populations. More recently, there has been growing interest in marine algae and their constituents as functional foods and nutraceuticals with potential health benefit effects as sources of antioxidant to reduce the risk of neurodegenerative diseases. Marine algae are an important source of bioactive ingredients that can be applied to many aspects of processing healthier foods and developing functional neuroprotective foods.

In addition, the wide diversity of marine algae and numerous undiscovered unique metabolites present in marine algae are interesting sources to increase numbers of novel drugs against neurodegenerative diseases. However, large-scale human studies are required to identify the prophylactic and therapeutic neuroprotective effect of marine algae.

## 4. Conclusions

In conclusion, marine algae are a valuable source of neuroprotective agents and could be introduced for the preparation of novel functional ingredients in pharmaceuticals and functional foods as a good approach for the treatment and or prevention of neurodegenerative disease. Marine algae can be suggested as an alternative source to synthetic ingredients that can contribute to neuroprotection by being a part of pharmaceuticals and functional foods. Furthermore, the wide range of biological activities associated with natural compounds derived from marine algae such as phlorotannins, alginates, fucoidan, sargaquinoic acid, SPs and carotenoids increase the potential to expand the neuroprotective effects and health beneficial value of marine algae in the pharmaceutical industry. Until now, most of the biological and neuroprotective activities of marine algae and its natural compounds have been observed *in vitro* or in mouse model systems. Therefore, further research studies are needed in order to investigate marine algae neuroprotective activities in human subjects and further in large-scale controlled studies.

## Figures and Tables

**Table 1.** Acetylcholinesterase inhibitory activities of several marine algae.

**Marine algae**	**Extracts/Compounds**	**IC_50_**	**Ref**
*Caulerpa racemosa*	MeOH extracts	5.5 mg mL^−1^	[70]
*Codium capitatum*	MeOH extracts	7.8 mg mL^−1^	[70]
*Ulva fasciata*	MeOH extracts	4.8 mg mL^−1^	[70]
*Halimeda cuneata*	MeOH extracts	5.7 mg mL^−1^	[70]
*Amphiora ephedraea*	MeOH extracts	5.1 mg mL^−1^	[70]
*Amphiora bowerbankii*	MeOH extracts	5.3 mg mL^−1^	[70]
*Dictyota humifusa*	MeOH extracts	4.8 mg mL^−1^	[70]
*Hypnea valentiae*	MeOH extracts	2.6 mg mL^−1^	[71]
*Padina gymnospora*	MeOH extracts	3.5 mg mL^−1^	[71]
*Ulva reticulate*	MeOH extracts	10 mg mL^−1^	[71]
*Gracilaria edulis*	MeOH extracts	3 mg mL^−1^	[71]
*Ecklonia stolonifera*	EtOH extracts	108.11 μg mL^−1^	[72]
*Ecklonia stolonifera*	24–hydroperoxy–24–vinylcholesterol	389.1 μM	[72]
*Ecklonia stolonifera*	Eckstolonol	42.66 μM	[72]
*Ecklonia stolonifera*	Eckol	20.56 μM	[72]
*Ecklonia stolonifera*	Phlorofucofluoroeckol–A	4.89 μM	[72]
*Ecklonia stolonifera*	Dieckol	17.11 μM	[72]
*Ecklonia stolonifera*	2–phloroeckol	38.13 μM	[72]
*Ecklonia stolonifera*	7–phloroeckol	21.11 μM	[72]
*Ishige okamurae*	MeOH extracts	163.07 μM	[73]
*Ishige okamurae*	EtOAc extracts	137.25 μM	[73]
*Ishige okamurae*	6,6′–bieckol	46.42 μM	[73]

IC_50_ values for eserine and galanthamine were 0.004 μg mL^−1^ and 0.0007 mg mL^−1^, respectively.
